# Role of Ultrasound and Fetal MRI in the Prenatal Assessment of Congenital Cytomegalovirus Infection: A Systematic Review

**DOI:** 10.3390/jcm15103645

**Published:** 2026-05-09

**Authors:** Katarzyna Stefanska, Krzysztof Berbeka, Dorota Madej, Piotr Witkowski, Magda Rybak-Krzyszkowska, Sambor Sawicki, Miriam Illa

**Affiliations:** 1 Department of Gynecology and Obstetrics, Medical University of Gdansk, 80-210 Gdańsk, Poland; 2 Ist Department of Obstetrics and Gynecology, Centre of Postgraduate Medical Education, 01-813 Warszawa, Poland; 3 Department of Obstetrics and Perinatology, University Hospital, 31-501 Krakow, Poland; 4 Hi-Gen Centrum Medyczne, 31-322 Krakow, Poland; 5 BCNatal, Fetal Medicine Research Center (Hospital Clinic and Hospital Sant Joan de Déu), Universitat de Barcelona, 08007 Barcelona, Spain; 6 Institut de Recerca Sant Joan de Déu (IRSJD), Fundació Sant Joan de Déu, 08014 Barcelona, Spain; 7 Spanish Network in Maternal, Neonatal, Child and Developmental Health Research (RICORS-SAMID, RD24/0013/0004), Instituto de Salud Carlos III, 28040 Madrid, Spain

**Keywords:** CMV, congenital CMV infection, magnetic resonance imaging, obstetric ultrasound, neurosonography

## Abstract

**Background:** Congenital cytomegalovirus (CMV) infection is the most common congenital infection worldwide and a leading cause of neurodevelopment impairment. Prenatal imaging plays a central role in diagnosis and prognostic assessment. **Methods:** We conducted a systematic review of studies evaluating the role of ultrasound, including dedicated fetal neurosonography and magnetic resonance imaging (MRI) in the prenatal detection and assessment of CMV infection. PubMed, Web of Science, and Scopus were searched for relevant studies, and data were synthesized focusing on detected abnormalities and the incremental diagnostic value of MRI. **Results:** Fifty-nine studies were included. Dedicated neurosonography (NSG) was the primary modality for detecting CMV-related fetal brain abnormalities, particularly ventriculomegaly and cortical malformations. Fetal MRI provided additional diagnostic information, mainly through improved visualization of the brain parenchyma, allowing better detection of white matter abnormalities, migrational disorders and posterior fossa involvement, especially when performed later in gestation. **Conclusions:** Dedicated neurosonography remains the cornerstone for the evaluation of suspected congenital fetal CMV infection. Fetal MRI represents a complementary tool that can confirm, refine or extend ultrasound findings and may improve prognostic assessment when additional or subtle brain abnormalities are suspected.

## 1. Introduction

Congenital cytomegalovirus (CMV) infection is the most common congenital infection worldwide, affecting approximately 0.5–2.5% of all live births [1]. It represents the leading non-genetic cause of sensorineural hearing loss and an important contributor to neurodevelopmental impairment, including motor deficits, intellectual disability, epilepsy, and visual abnormalities [2,3]. Even in infants who are asymptomatic at birth, late-onset sequelae—particularly hearing loss—may develop during early childhood, resulting in a substantial long-term burden for affected individuals, families, and healthcare systems [3,4].

The virus is transmitted through direct or indirect contact with human secretions, such as urine, saliva, vaginal secretions, semen, breast milk, and blood products, and transplanted organs. Virus shedding is the longest in primary infection and is the leading cause of congenital infection. The clinical impact of congenital CMV infection is strongly influenced by the timing of maternal infection. Transmission occurring during the periconceptional period or the first trimester of pregnancy is associated with the highest risk of severe fetal brain injury and adverse neurodevelopmental outcomes, reaching up to 30–40% in early gestation [5,6]. In contrast, although the rate of maternal–fetal transmission increases with advancing gestational age and may exceed 70% in the third trimester, infections acquired later in pregnancy are more frequently associated with milder or subclinical disease [5]. This inverse relationship between transmission rate and disease severity reflects the vulnerability of the developing fetal brain during early neurogenesis and neuronal migration and represents a key determinant of prognosis in congenital CMV infection [6].

Prenatal diagnosis of congenital CMV infection remains challenging. There is a limited number of worldwide guidelines for CMV screening in pregnant women and universal antenatal screening is not currently recommended in most countries [7,8]. As a result, many congenital infections remain undetected at birth, particularly in asymptomatic infants, and are only diagnosed later when clinical sequelae become apparent. This lack of systematic screening complicates the establishment of a clear temporary link between maternal infection, fetal involvement, and postnatal disease expression. In parallel, growing knowledge regarding the mechanisms of fetal injury, parental expectations following a diagnosis of intrauterine infection, and emerging evidence from studies on antiviral therapies and immunoglobulins has increased interest in preventive and therapeutic strategies aimed at reducing fetal morbidity [9]. In this context, prenatal imaging plays a central role in the detection and evaluation of fetal CMV infection. Ultrasound, including dedicated fetal neurosonography, remains the first-line imaging modality and is widely used to identify both cerebral and extracerebral manifestations of infection [10]. Typical central nervous system findings include ventriculomegaly, intracranial calcifications, periventricular lesions, cortical malformations, cerebellar abnormalities, microcephaly, and white matter abnormalities [6,11,12,13,14,15,16]. Fetal magnetic resonance imaging (MRI) has emerged as a complementary tool, particularly in cases with normal or equivocal ultrasound findings or when complex central nervous system abnormalities are suspected. MRI allows improved visualization of brain parenchyma, cortical development, white matter abnormalities, and posterior fossa structures, especially when performed later in gestation [13]. However, normal prenatal ultrasound and MRI findings do not exclude the risk of postnatal sequelae, particularly sensorineural hearing loss, especially following early maternal infection [6,12,16].

Despite the increasing use of prenatal imaging, significant variability exists in the reported diagnostic performance and prognostic value of ultrasound and fetal MRI in congenital CMV infection. Differences in imaging protocols, timing of examinations, operator expertise, and outcome assessment contribute to ongoing uncertainty in prenatal counseling. Despite the growing number of systematic reviews addressing prenatal imaging in congenital CMV infection, existing syntheses have largely focused on single imaging modalities or selected aspects of diagnostic assessment. In particular, prior reviews have typically examined either the role of ultrasonography as a screening and monitoring tool or the utility of fetal MRI in the evaluation of the central nervous system, often without direct comparison between these techniques or integration within a unified diagnostic framework. Furthermore, many of these studies have not included a systematic assessment of study quality or risk of bias. The present systematic review extends the existing body of evidence by providing a parallel evaluation of both ultrasonography and fetal MRI in the prenatal assessment of congenital CMV infection, emphasizing their complementary roles, differences in diagnostic sensitivity, and the clinical relevance of detected abnormalities. In addition, the application of a structured quality assessment enables a more critical appraisal of the available evidence and supports a more robust translation of findings into clinical practice.

## 2. Materials and Methods

### 2.1. Eligibility Criteria

Predefined inclusion and exclusion criteria were established prior to the literature search. Studies were eligible for inclusion if they evaluated the role of prenatal ultrasonography, including dedicated fetal neurosonography, and/or fetal magnetic resonance imaging (MRI) in the assessment of suspected or confirmed congenital cytomegalovirus (CMV) infection. Eligible studies were required to report prenatal imaging findings in fetuses with suspected or confirmed congenital CMV infection and to provide information on cerebral and/or extracerebral abnormalities detected by ultrasound and/or MRI.

Studies were excluded if they focused exclusively on maternal infection, serological diagnosis, treatment, prevention, or postnatal imaging without reporting prenatal imaging findings. Review articles, editorials, conference abstracts, and case reports with insufficient imaging description were excluded. Only studies published in English between January 2003 and October 2025 were considered.

### 2.2. Information Sources

A systematic literature search was conducted using the following electronic databases: PubMed, Web of Science, and Scopus. The last search was performed on 1 October 2025.

### 2.3. Search Strategy

The search strategy was designed to identify studies evaluating prenatal imaging in the context of congenital CMV infection. Search terms included combinations of keywords related to cytomegalovirus infection, pregnancy, prenatal ultrasound, fetal neurosonography, and fetal magnetic resonance imaging. Keywords were combined using Boolean operators (AND/OR) and applied to titles and abstracts. The reference lists of all included articles were manually screened to identify additional relevant studies.

A summary of the search terms used for each database is provided in Table 1.

### 2.4. Study Selection

**Figure 1:**
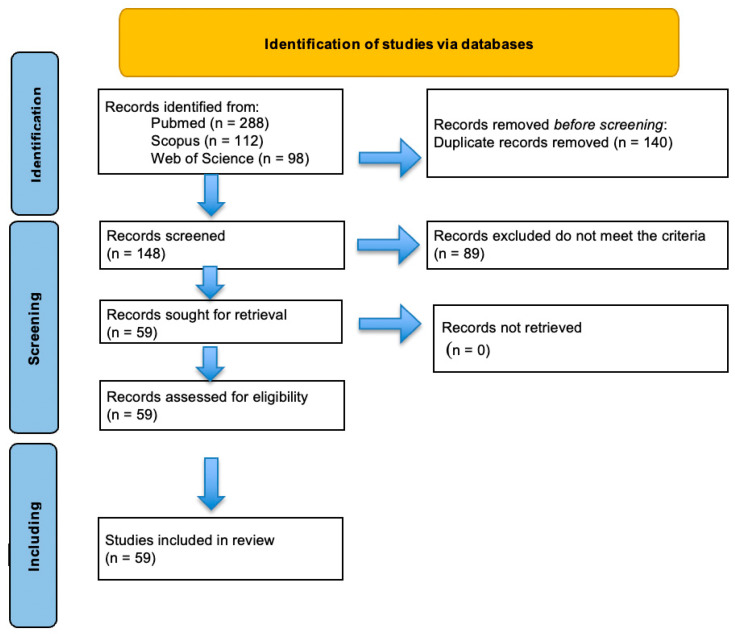
PRISMA flow sheet highlighting the selection process for finalizing the articles.

This systematic review was conducted in accordance with the Preferred Reporting Items for Systematic Reviews and Meta-Analyses (PRISMA) guideline [17]. Study selection was performed in two stages. First, titles and abstracts were screened to exclude clearly irrelevant studies. Second, full-text articles were assessed for eligibility based on the predefined inclusion and exclusion criteria. The selection process was conducted independently, and any disagreements were resolved through discussion and consensus among the authors. The study selection process is summarized in a PRISMA flow diagram (Figure 1).

Following application of the inclusion and exclusion criteria, a total of 59 studies were included in the final review. The main characteristics of the included studies, including study design, study population, imaging modality, and reported prenatal imaging findings, are summarized in Supplementary Table S1. The protocol for this systematic review was prospectively registered in the PROSPERO database (International Prospective Register of Systematic Reviews; registration number: CRD420261339321. This enhances methodological transparency and minimizes the risk of reporting bias.

### 2.5. Data Extraction and Synthesis

Data extracted from the included studies comprised study characteristics (author, year, study design), population characteristics, imaging modality (prenatal ultrasound and/or fetal MRI), reported fetal brain abnormalities (e.g., ventriculomegaly, intracranial calcifications, cortical malformations, white matter abnormalities), and reported neonatal or postnatal outcomes, including sensorineural hearing loss and neurodevelopmental impairment. Given the heterogeneity of study designs, imaging protocols, and outcome measures, a qualitative synthesis of the results was performed.

### 2.6. Risk of Bias

The methodological quality and risk of bias of the included studies were assessed using the QUADAS-2 tool, which is specifically designed for systematic reviews of diagnostic accuracy studies. The QUADAS-2 tool evaluates four key domains: patient selection, index test, reference standard, flow and timing. The assessment was performed independently by two reviewers. Any disagreements were resolved through discussion, and if consensus could not be reached, a third reviewer was consulted. The methodological quality of the included studies substantially influences the interpretation of the findings of this review. Variability in study design and patient selection, particularly the inclusion of high-risk populations from tertiary referral centers, may lead to an overestimation of the prevalence of fetal abnormalities and, consequently, inflated estimates of diagnostic performance. Heterogeneity in imaging protocols and timing further affects comparability across studies. The use of advanced neurosonography and the performance of fetal MRI at later gestational ages increase the detection of subtle cortical and white matter abnormalities, potentially contributing to higher reported sensitivity in studies with more standardized and optimized methodologies. Additionally, the lack of consistent blinding and the potential for interobserver variability introduce a risk of interpretation bias. Knowledge of prior ultrasound findings during MRI assessment may lead to confirmation bias and overestimation of the incremental value of MRI. Overall, these factors suggest that the reported complementary role of fetal MRI should be interpreted with caution. Evidence derived from prospective studies with standardized imaging protocols and blinded assessment is likely to provide more reliable and generalizable estimates.

## 3. Results

### 3.1. Frequency of US Examination/NSG and MRI in Pregnancy

Across the included studies, fetal central nervous system (CNS) abnormalities were commonly assessed using prenatal ultrasound, reflecting the central role of imaging in the evaluation of fetuses at risk of congenital CMV infection. Although some patients were identified as high risk because of maternal infection or other clinical factors, many CNS abnormalities were reported in fetuses without known predisposing conditions. Routine mid-trimester ultrasound examinations, typically performed between 20 and 24 weeks of gestation, were described across studies as the primary time point for initial detection of CNS abnormalities [18,19,20].

Most studies identified targeted neurosonography as the primary imaging modality [21,22,23]. A suspected congenital CMV was also reported as a frequent indication for a targeted neurosonography during follow-up in referral centers, typically performed by experienced operators, with transvaginal ultrasound described as particularly useful for improved resolution of the periventricular parenchyma and assessment of cortical development in cephalic presentation [24].

In fetuses with confirmed congenital CMV infection, several studies reported serial targeted neurosonography follow-up examinations, often performed at intervals of approximately 2–3 weeks, with particular emphasis on detailed assessment of the fetal brain [25]. In cases of suspected maternal CMV infection without confirmed fetal involvement, repeated ultrasound surveillance every 2–3 weeks was also described, aiming to detect delayed-onset cerebral or extracerebral manifestations [25]. When antenatal ultrasound examination of the fetal brain was reported as normal in fetuses with confirmed congenital CMV infection, early neuropsychological outcome was generally favorable in several series, although this did not exclude all adverse outcomes, such as sensorineural hearing loss. Despite normal prenatal US, infection with clinical manifestations and abnormal neurodevelopmental outcome were observed in 1.5% and 3.1% of fetuses, respectively, while hearing impairment occurred in 6.5% [26,27].

Fetal magnetic resonance imaging (MRI) was reported as an adjunctive imaging modality in a subset of studies, most commonly performed in the late second or third trimester. MRI examinations were typically conducted between 28 and 32 weeks of gestation and, in some studies, repeated later in pregnancy to further characterize brain involvement or to assess lesion progression [25]. Across studies, ultrasound and MRI were described as complementary imaging modalities for investigation of the fetal brain [28]. When both modalities were performed in the third trimester in a fetus known to be infected with CMV, their combined performance was reported to identify the majority of CMV-related CNS lesions described in the literature [29]. When both ultrasound and MRI of the fetal brain are normal prenatally, the neonatal outcome is generally good, and the same may be true for a normal ultrasound examination with only subtle findings on MRI, e.g., isolated T_2_-weighted white matter hyperintense signal (WMHS) [6,29]. However, normal prenatal imaging findings were not considered predictive of hearing outcome, as sensorineural hearing loss may still occur despite normal prenatal ultrasound and MRI [29].

### 3.2. Ultrasound for Fetal CMV Infection

Across the included studies, prenatal ultrasound was reported as the primary imaging modality for the assessment of fetuses with suspected or confirmed congenital CMV infection. Ultrasound findings were used to evaluate both central nervous system (CNS) involvement and extracerebral manifestations associated with fetal or placental infection. These abnormalities were attributed to inflammatory, destructive, and obstructive processes affecting the developing brain and were reported to have a direct impact on fetal prognosis [6,10]. In addition, several studies described increased echogenicity of visceral organs and placental abnormalities in association with intrauterine CMV infection [30,31].

#### 3.2.1. Intracranial Findings

**Figure 2:**
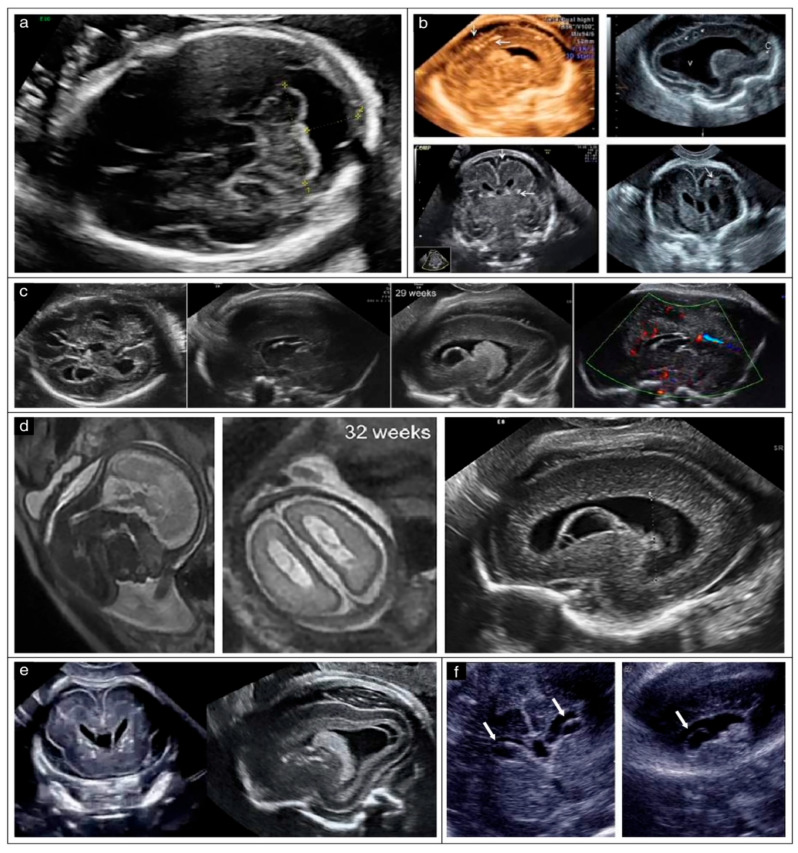
Ultrasonographic cranial features typical of CMV infection [10]. (**a**) Megacisterna magna, (**b**) intracranial calcifications, (**c**,**d**) ventriculomegaly, germinolytic cysts, agenesis of corpus callosum and intraventricular adhesions, (**c**,**f**) periventricular cystic changes, (**d**) lissencephaly, (**e**) cerebral calcifications and periventricular cysts, and (**f**) subependymal cysts.

Typical cranial ultrasound features reported in fetuses with congenital CMV infection included ventriculomegaly, intraventricular adhesions, enlargement of the cisterna magna, intracranial calcifications, periventricular cystic changes, abnormalities of the corpus callosum, and malformations of cortical development such as lissencephaly [6,10]. Among these findings, increased periventricular echogenicity, cystic changes, and intracranial calcifications were consistently described across studies as characteristic imaging features of congenital CMV infections [1,32]. See Figure 2 where typical CMV images are presented.

Among all these intracranial abnormalities, ventriculomegaly was the most consistently reported and frequently the earliest ultrasound finding in fetuses with congenital CMV infection. It was commonly described as the initial abnormality prompting referral for targeted neurosonography and closer imaging follow-up. Both severe forms along with progression over time have been associated with a higher likelihood of additional central nervous system abnormalities and less favorable outcomes [22,33].

Abnormalities of the corpus callosum and posterior fossa were also reported as part of the imaging spectrum of CMV fetopathy. Disruption of the progenitor cell pool and altered neuronal migration have been proposed as underlying mechanism leading to callosal agenesis, hypoplasia, or dysplasia, as well as pontocerebellar or cerebral hypoplasia [29]. In this context, Krajden Haratz K. et al. described the rate and pattern of callosal injury in cytomegalovirus (CMV) fetopathy, highlighting that callosal involvement may occur even in late infection and contributes to the wide spectrum of neurological manifestations observed in congenital CMV infection [32].

Other CNS manifestation of CMV fetal infection, such as ocular abnormalities beyond microphthalmia or cataracts, were rarely reported prenatally and were described as difficult to detect using fetal ultrasound. Findings such as optic nerve hypoplasia or atrophy were considered to be beyond the diagnostic capabilities of routine prenatal sonography in most studies [29].

Across the included studies, ultrasound findings were frequently stratified according to severity. Severe central nervous system abnormalities included ventriculomegaly (≥15 mm), periventricular hyperechogenicity, hydrocephalus, microcephaly, and destructive brain lesions such as porencephaly and lissencephaly. Mild abnormalities comprised mild ventriculomegaly, subependymal cysts, intracerebral calcifications, and lenticulostriate vasculopathy. Table 2 summarizes the findings depending on the severity.

Beyond the detection of structural abnormalities, ultrasound was also used to monitor the temporal evolution of fetal brain findings. The presence of periventricular echogenic halo was reported as a marker of white matter involvement, with variable clinical outcomes described across studies [34]. Focal germinal matrix changes, often associated with lenticulostriate vasculopathy, were predominantly reported in late gestation [15].

#### 3.2.2. Placenta and Extracranial Findings

In addition to central nervous system involvement, several studies reported placental and extracranial ultrasound findings associated with congenital CMV infection, reflecting the systemic nature of fetal involvement [10,24,35]. Across studies, these findings were not considered diagnostic in isolation but were reported as supportive features in the prenatal assessment of suspected fetal infection. Extracerebral findings reported across studies comprised hyperechogenic bowel, hepatosplenomegaly, fetal growth restriction, abnormal amniotic fluid volume, ascites, hydrops, and placentomegaly [22,33,36]. These findings were reported either as isolated manifestations or in combination with placental and cerebral abnormalities. Several studies reported that extracerebral manifestations often preceded cerebral abnormalities, with CNS involvement becoming apparent later in gestation as the infection evolved [22,33,36].

Placental involvement was primarily reported in association with maternal viremia and placental infection. Placentomegaly was the most frequently described placental abnormality and was commonly defined as a placental thickness greater than 40 mm, although some authors reported progressive placental thickening according to gestational age between 16 and 36 weeks [10]. Placental calcifications were also described in a subset of cases.

Several studies also reported fetal growth restriction as a possible manifestation of congenital CMV infection, resulting from fetal involvement, placental infection, or both. In some cases, fetal growth restriction was described as an isolated finding, independent of abnormal placental or fetal Doppler parameters, supporting its consideration as a potential indication for CMV screening in fetuses below the 5th centile [33].

Table 3 summarizes the reported frequency of placental and extracranial ultrasound findings across studies [24,35]. For comparative purposes, the table also includes the frequency of selected cerebral ultrasound abnormalities reported in the same studies, such as ventriculomegaly, microcephaly, intracranial calcifications, hydrocephalus, hyperechogenic periventricular halo, subependymal cysts, and cortical development abnormalities, including heterotopias or polymicrogyria. These cerebral findings have been described in detail in previous sections and are included here to provide an integrated overview of the spectrum and relative frequency of ultrasound-detected abnormalities in congenital CMV infection.

### 3.3. MRI for Fetal CMV Infection

Across the included studies, fetal magnetic resonance imaging (MRI) was reported as a complementary imaging modality for the assessment of fetal brain involvement in congenital CMV infection. MRI was most commonly performed when ultrasound findings were inconclusive or suggested complex central nervous system (CNS) abnormalities [22,37]. Across studies, inconclusive ultrasound assessment was not only related to the complexity of fetal brain abnormalities but also to technical limitations, with maternal obesity, abdominal scarring, and oligohydramnios reported as factors impairing ultrasound image quality in selected cases [38]. While several studies initially evaluated the contribution of fetal MRI in fetuses with isolated CNS malformations undergoing multiplanar brain assessment [39,40,41,42], a smaller number specifically investigated its role in congenital CMV infection. These studies frequently reported small sample sizes, heterogeneous imaging protocols, and inclusion of cases with pre-existing ultrasound abnormalities, which limited the generalizability of their findings and direct comparisons across studies [27].

**Figure 3:**
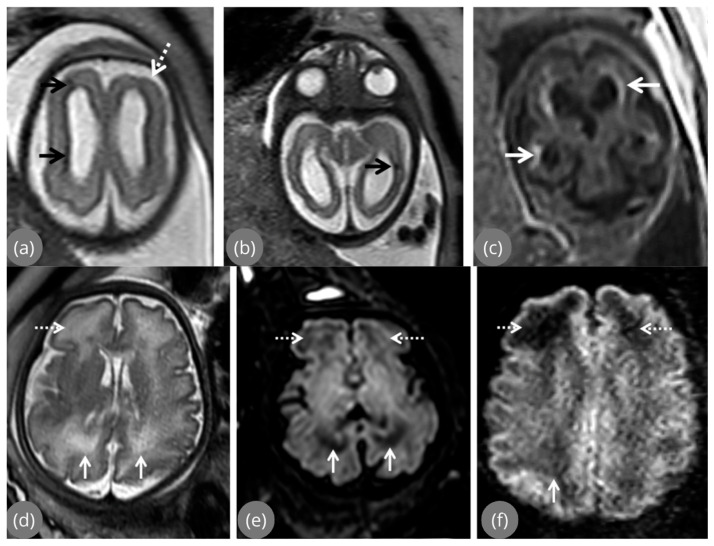
MRI features typical of CMV infection [13]. (**a**,**b**) focal signal anomaly—black arrows, (**a**) frontal polymicrogyria—white dashed arrow, (**c**) calcifications—white arrows, (**d**) white matter hyperintensities in the frontal—white dashed arrows and parieto-occipital region—white arrows, (**e**) T2w echo planar imaging-FLAIR hypointensity—white/dashed arrows, and (**f**) low SI on the zoom diffusion weighted image—white/dashed arrows.

MRI-detected abnormalities reported in fetuses with congenital CMV infection included ventriculomegaly, periventricular leukomalacia, porencephaly, schizencephaly, microcephaly, heterotopias, and malformations of cortical development [35], with several studies reporting that fetal MRI allows detailed assessment of brain parenchyma, cortical organization, posterior fossa structures, white matter signal characteristics, and deep brain structures involved in congenital CMV infection. Regarding heterotopias and polymicrogyria, these abnormalities were predominantly detected by MRI, particularly when limited to the temporal lobes, and were therefore reported as potentially occult on ultrasound examination [13,33,35]. Ventriculomegaly, frequently described as the most common and earliest abnormality on prenatal ultrasound, was further characterized by MRI in several studies in terms of associated parenchymal damage, cortical malformations, or white matter involvement [13,33,35] (see next section). See Figure 3 where typical CMV images were presented.

The incremental diagnostic value of fetal MRI appears particularly relevant in fetuses with confirmed congenital CMV infection and normal neurosonography. In a large multicenter cohort including 95 fetuses with normal dedicated neurosonography, additional structural CNS anomalies were detected exclusively on fetal MRI in 10.5% of cases. The MRI-only findings comprised mainly malformations of cortical development (40%), destructive encephalopathy of the white matter (20%), intracranial calcifications involving the germinal matrix or basal ganglia (10%), and complex CNS anomalies (30%). Notably, no ventricular, periventricular, midline or posterior fossa anomalies were detected exclusively by MRI in this cohort [43]. In the same cohort, CMV viral load in amniotic fluid was the only independent predictor of detecting additional CNS anomalies on fetal MRI in cases with normal neurosonography, both as a continuous variable and using a cut-off of >100,000 copies/mL. This finding suggests that virological markers may help identify a subgroup of fetuses with normal neurosonography in whom fetal MRI is more likely to provide additional diagnostic information.

The spectrum of MRI abnormalities reported across studies in congenital CMV infection reflected both the timing and the extent of fetal brain injury. Early insults were associated with germinal matrix involvement, neuronal loss, and diffuse disturbances of neuronal migration, whereas later insults were more frequently associated with focal or subtler abnormalities [13,33,44]. Lissencephaly was most commonly reported following early insults occurring before 16–18 weeks of gestation, whereas polymicrogyria was more frequently described in association with injuries occurring between 18 and 24 weeks. Infections occurring during the third trimester were more often associated with preserved gyral patterns, occasionally accompanied by diffuse or heterogeneous white matter signal abnormalities on T2-weighted imaging [13,30]. Both T1- and T2-weighted sequences were used across studies to characterize these findings [33].

Diffusion-weighted imaging (DWI) and apparent diffusion coefficient (ADC) measurements were reported in a limited number of studies and were described as providing quantitative information on fetal brain maturation and tissue integrity in selected cases [23]. However, interpretation of isolated white matter signal abnormalities, particularly T2-weighted hyperintensity in the third trimester, was reported to be variable and subjective, with inconsistent associations with postnatal outcome across studies [13,29,37,39,40,41,42,43,45,46].

In addition to more common abnormalities, several studies reported rarer MRI findings in fetuses with congenital CMV infection, including hippocampal dysplasia and cerebellar hypoplasia [46]. These abnormalities were most frequently described in examinations performed during the third trimester, when advanced gyral development allowed improved visualization of cortical and posterior fossa structures [46].

Overall, fetal MRI was consistently described as a complementary technique to prenatal ultrasound in the assessment of congenital CMV infection, providing additional anatomical detail in selected cases, particularly with respect to cortical development, white matter involvement, and posterior fossa abnormalities [22,37]. When dedicated neurosonography identifies clear structural CNS abnormalities in fetuses with congenital CMV infection, fetal MRI is mainly used to further characterize lesion extent and parenchymal involvement. In these cases, MRI usually refines the anatomical definition rather than substantially modifying the overall diagnostic or prognostic assessment [43].

### 3.4. Correlation Between Prenatal Imaging Findings and Neonatal Outcome

Across the included studies, prenatal ultrasound and fetal magnetic resonance imaging (MRI) findings were consistently reported to correlate with neonatal and postnatal outcomes in fetuses with congenital CMV infection. The prognostic value of prenatal imaging was shown to depend primarily on the severity, extent, and progression of central nervous system (CNS) abnormalities over time, rather than on the presence of isolated findings at a single examination [4,21,33,35,46,47,48,49,50,51]. Several studies emphasized that the pattern of imaging abnormalities was closely related to the timing of fetal brain injury, which in turn reflected the gestational age at maternal infection.

Severe and progressive CNS abnormalities identified on prenatal imaging were repeatedly associated with adverse neonatal and long-term neurodevelopmental outcomes. Second-trimester ultrasound findings such as progressive or marked ventriculomegaly, extensive periventricular hyperechogenicity, hydrocephalus, and microcephaly were reported as indicators of widespread brain involvement and were frequently linked to poor neurological prognosis, including motor impairment, epilepsy, and cognitive delay [21,33,35,44,47,48,49,50,51]. Destructive brain lesions, including porencephaly, lissencephaly, and extensive cortical malformations, were also consistently associated with severe postnatal outcomes and were predominantly reported in cases of early maternal infection.

In addition to lesion severity, the temporal evolution of imaging findings was described as a relevant prognostic factor. Several studies reported that abnormalities initially detected as mild or focal could progress over time, with subsequent appearance of additional CNS lesions later in gestation. This progressive pattern was associated with a higher likelihood of adverse outcomes compared with stable or non-progressive findings [33,35,44]. Conversely, isolated abnormalities that remained stable throughout pregnancy were more often associated with favorable early outcomes.

Isolated or mild prenatal imaging findings were generally associated with a more favorable neonatal course. These findings included mild to moderate ventriculomegaly (<15 mm), subependymal cysts, lenticulostriate vasculopathy, isolated intracranial or parenchymal calcifications, and limited white matter abnormalities [21,35,52,53]. Several studies reported that fetuses with such isolated findings were frequently asymptomatic at birth and demonstrated normal early neurodevelopment. However, even in this group, a non-negligible risk of delayed-onset sequelae was described, particularly sensorineural hearing loss, which affected up to 13.5% of asymptomatic newborns and could occur independently of the presence or absence of prenatal CNS abnormalities [35,52,53]. In the same line, the prognostic significance of subtle or isolated MRI findings, particularly isolated white matter signal abnormalities detected in late gestation, remained inconsistent across studies, with variable associations with postnatal neurodevelopmental outcome [13,29,37,39,40,41,42,43,45,46].

In this context, fetal MRI was reported to provide additional prognostic information in selected cases, particularly when prenatal ultrasound findings were normal or equivocal. As explained previously, additional CNS abnormalities were detected exclusively by fetal MRI in approximately 10% of fetuses with congenital CMV infection and normal neurosonography [43]. The MRI-only abnormalities detected in this cohort predominantly involved cortical development and early white matter injury, which are known to reflect earlier stages of CMV-related brain damage. However, the prognostic significance of these isolated MRI findings was heterogeneous across studies and did not uniformly translate into adverse postnatal outcome.

Finally, normal prenatal imaging findings were also reported in a proportion of fetuses with confirmed congenital CMV infection. Across studies, normal ultrasound and MRI examinations were generally associated with a favorable short-term outcome. Nevertheless, multiple reports emphasized that normal prenatal imaging did not reliably exclude the risk of later neurological or auditory impairment, underscoring the limitations of prenatal imaging for the prediction of long-term outcome, particularly following early maternal infection [21,35,52].

In addition to imaging findings, several studies highlighted that the prognostic performance of prenatal assessment in congenital CMV infection was improved when imaging data were interpreted together with laboratory markers. In this context, CMV viral load in amniotic fluid emerged as a relevant modifier of imaging findings, being independently associated with the detection of CNS abnormalities on fetal MRI at later gestational ages. These observations support an integrated approach combining virological and imaging data for prenatal risk stratification [43].

### 3.5. Proposed Management Algorithm

**Figure 4:**
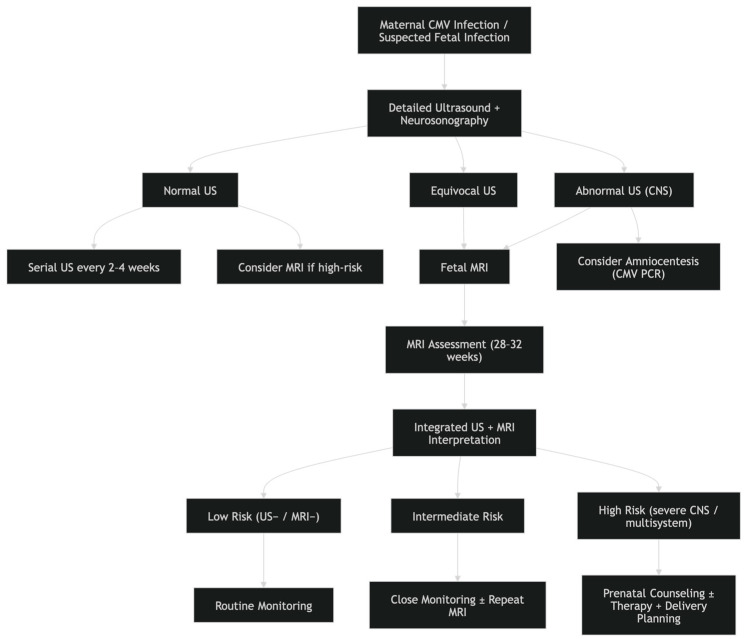
Diagnostic algorithm for congenital CMV.

Based on current knowledge, a proposed diagnostic algorithm for congenital CMV has been developed. It is presented in Figure 4.

## 4. Discussion

This systematic review highlights the central role of prenatal imaging in the evaluation and management of congenital cytomegalovirus (CMV) infection, while also underscoring its limitations for individual prognostic prediction. Across the available literature, prenatal ultrasound and fetal magnetic resonance imaging (MRI) emerge as complementary tools, whose clinical value depends primarily on the severity, extent, temporal evolution, and timing of fetal brain involvement rather than on the presence of isolated imaging findings. Importantly, imaging findings must be interpreted within a dynamic framework, integrating gestational age, longitudinal evolution, and additional clinical and laboratory information. Previous systematic reviews on prenatal imaging in congenital CMV infection have generally reported consistent findings regarding the value of both ultrasonography and fetal MRI, while differing in the extent to which each modality was emphasized. Most earlier studies have highlighted ultrasonography as the primary screening and follow-up tool, with fetal MRI considered mainly as a complementary technique for detailed assessment of suspected central nervous system abnormalities. In line with these reports, our findings confirm the complementary roles of both modalities; however, our review provides a more integrated synthesis of the available evidence, directly comparing their diagnostic contributions within the same analytical framework. This approach allows for a clearer understanding of the relative strengths and limitations of each imaging technique in the prenatal evaluation of congenital CMV infection.

A consistent observation across studies is the strong influence of the timing of maternal infection on both imaging patterns and outcome. Early maternal infection, particularly during the periconceptional period or first trimester, is more frequently associated with severe and diffuse central nervous system (CNS) abnormalities, including cortical malformations, destructive lesions, and microcephaly, which carry a high risk of adverse neurodevelopmental outcome. In contrast, infections occurring later in gestation tend to result in more focal, subtle, or even absent prenatal imaging abnormalities, with greater variability in postnatal outcome [3]. This timing-dependent pattern partly explains why apparently reassuring prenatal imaging findings may coexist with later-onset sequelae, particularly following early maternal infection, and represents a key challenge in prenatal counseling.

Prenatal ultrasound remains the first-line imaging modality in suspected fetal CMV infection and plays a pivotal role in longitudinal fetal assessment. Its main strengths include widespread availability, feasibility for repeated examinations, and reliable detection of severe and progressive CNS abnormalities, which consistently correlate with poor neonatal and long-term outcomes. Progressive ventriculomegaly, microcephaly, hydrocephalus, and extensive periventricular abnormalities are among the ultrasound findings most strongly associated with adverse prognosis. In addition, placental and extracranial ultrasound findings, such as placentomegaly, fetal growth restriction, hyperechogenic bowel, ascites, or hepatosplenomegaly, reflect the systemic nature of CMV infection and may support suspicion of fetal involvement, although they lack diagnostic specificity when considered in isolation.

Despite its central role, prenatal ultrasound has well-recognized limitations, particularly in the detection of subtle cortical abnormalities and white matter involvement, and its diagnostic performance may be further impaired by maternal and technical factors such as obesity, abdominal scarring, and oligohydramnios. In this context, fetal MRI has emerged as a valuable complementary imaging modality. MRI provides improved visualization of cortical development, white matter signal abnormalities, posterior fossa structures, and deep brain anatomy. It may also detect additional CNS abnormalities not apparent on ultrasound in a small proportion of fetuses with congenital CMV infection and normal neurosonography. These MRI-only findings encompass a broad and heterogeneous spectrum, and their independent prognostic impact is not consistently established across studies. Consequently, the clinical relevance of such findings should be interpreted with caution and within the broader context of gestational timing, lesion severity, and longitudinal imaging evolution. Its added value, therefore, is not uniform across all cases and appears to be influenced by the severity and complexity of abnormalities detected on ultrasound. In clinical practice, fetal MRI may provide reassurance in cases of mild or moderate ventriculomegaly, while in severe or progressive ventriculomegaly it more frequently identifies associated parenchymal, cortical, or vascular abnormalities that contribute to prognostic assessment.

The correlation between prenatal imaging findings and outcome is one of the most clinically relevant aspects of CMV infection. Across studies, severe and progressive CNS abnormalities detected on ultrasound or MRI are consistently associated with adverse neonatal and long-term neurodevelopmental outcomes. Conversely, isolated or mild imaging findings are generally associated with more favorable early outcomes. However, a key and clinically important message emerging from this review is that normal or near-normal prenatal imaging does not exclude the risk of later-onset sequelae. In particular, sensorineural hearing loss may occur in infants with normal prenatal ultrasound and MRI examinations, highlighting the inherent limitations of prenatal imaging for predicting all CMV-related outcomes.

Within this framework, fetal MRI appears to have a particularly high negative predictive value for severe structural brain injury and major neurological impairment when no abnormalities are detected, providing important reassurance in selected cases [48]. At the same time, subtle or isolated MRI abnormalities, especially mild white matter signal changes detected in late gestation, are associated with greater prognostic uncertainty and show inconsistent associations with postnatal outcome across studies. These findings emphasize the need for cautious interpretation of mild MRI abnormalities and for avoiding overestimation of their prognostic significance in prenatal counseling.

In this context, the role of fetal MRI in cases of confirmed congenital CMV infection with persistently normal neurosonography deserves particular consideration. Given the non-negligible proportion of MRI-only CNS abnormalities reported in such cases, and the limitations of ultrasound in detecting subtle cortical and early white matter involvement, some authors have advocated incorporating fetal MRI into longitudinal imaging follow-up even when neurosonography remains normal [44]. This approach may be particularly appropriate in tertiary referral centers with integrated fetal MRI expertise and multidisciplinary teams, where MRI findings can be interpreted within a comprehensive clinical, virological and imaging framework. In these settings, offering fetal MRI during the third trimester as part of routine follow-up in confirmed CMV infection may help refine risk assessment and counseling, while acknowledging that normal MRI findings do not fully exclude the risk of delayed-onset sequelae.

Across studies, virological markers have been reported to further refine prenatal risk stratification when interpreted alongside imaging findings. In particular, CMV viral load in amniotic fluid has been described as an independent predictor of MRI-detected CNS abnormalities at later gestational ages, supporting a complementary role of imaging and laboratory data in prenatal risk stratification. While these findings are promising, their clinical integration requires careful interpretation and further validation in prospective studies.

From a clinical perspective, prenatal counseling in congenital CMV infection remains challenging and must balance the need for clear information with the inherent uncertainty of individual prognosis. Imaging findings should be communicated as part of an integrated and dynamic assessment, taking into account the timing of infection, the severity and evolution of abnormalities, and the limitations of current diagnostic tools. While severe and progressive CNS abnormalities allow relatively confident identification of fetuses at high risk of adverse outcome, mild or absent imaging findings should be presented as reassuring but not definitive. In all cases, counseling should emphasize the importance of postnatal evaluation and long-term follow-up, particularly with respect to hearing and neurodevelopment.

Finally, several limitations of the available evidence should be acknowledged. The reviewed studies are characterized by substantial heterogeneity in study design, imaging protocols, timing of examinations, and duration of follow-up, as well as relatively small sample sizes. In addition, long-term neurodevelopmental and auditory outcomes are not uniformly reported. These limitations highlight the need for prospective, standardized studies with comprehensive postnatal follow-up to refine risk stratification and improve evidence-based prenatal counseling [22].

## 5. Conclusions

- Congenital cytomegalovirus infection remains a leading cause of neurodevelopmental morbidity, particularly sensorineural hearing loss, and is frequently underdiagnosed during pregnancy due to non-specific maternal presentation and limitations of routine screening strategies.
- Prenatal imaging plays a central role in the assessment of fetuses with suspected or confirmed CMV infection. Ultrasound represents the first-line modality, while fetal magnetic resonance imaging (MRI) provides complementary information in selected cases and may be considered as part of longitudinal follow-up in confirmed infections, particularly in specialized centers with integrated fetal MRI expertise.
- The prognostic value of prenatal imaging is primarily determined by the severity, extent, and progression of central nervous system abnormalities, as well as by the timing of maternal infection, rather than by isolated imaging findings.
- Severe and progressive prenatal imaging abnormalities are consistently associated with adverse neurodevelopmental outcomes, whereas mild or absent findings are generally associated with a more favorable prognosis, although they do not exclude the risk of delayed-onset sequelae, particularly hearing loss.
- Fetal MRI may offer a high negative predictive value for severe neurological impairment when no abnormalities are detected, but subtle MRI findings are associated with greater prognostic uncertainty and require cautious interpretation.
- Accurate prenatal counseling in congenital CMV infection should be individualized and based on an integrated, longitudinal assessment combining imaging findings, gestational timing, and virological data, with postnatal follow-up remaining essential.

## Figures and Tables

**Table 1:** Search strings for the literature databases.

Database	Search String
PubMed	((“Cytomegalovirus Infections” [Mesh] OR cytomegalovirus OR CMV OR cCMV OR “congenital cytomegalovirus”) AND (“Ultrasonography, Prenatal” [Mesh] OR ultrasound OR ultrasonography OR neurosonography OR “prenatal ultrasound”) AND (“Magnetic Resonance Imaging” [Mesh] OR MRI OR “fetal MRI” OR “magnetic resonance”) AND (fetus OR fetal OR foetal OR prenatal OR antenatal))
Web of Science	((“Cytomegalovirus Infections” [Mesh] OR cytomegalovirus OR CMV OR cCMV OR “congenital cytomegalovirus”) AND (“Ultrasonography, Prenatal” [Mesh] OR ultrasound OR ultrasonography OR neurosonography OR “prenatal ultrasound”) AND (“Magnetic Resonance Imaging” [Mesh] OR MRI OR “fetal MRI” OR “magnetic resonance”) AND (fetus OR fetal OR foetal OR prenatal OR antenatal))
Scopus	((“Cytomegalovirus Infections” [Mesh] OR cytomegalovirus OR CMV OR cCMV OR “congenital cytomegalovirus”) AND (“Ultrasonography, Prenatal” [Mesh] OR ultrasound OR ultrasonography OR neurosonography OR “prenatal ultrasound”) AND (“Magnetic Resonance Imaging” [Mesh] OR MRI OR “fetal MRI” OR “magnetic resonance”) AND (fetus OR fetal OR foetal OR prenatal OR antenatal))

**Table 2:** Intracranial ultrasound findings reported in association with congenital CMV infection stratified according to severity.

Severe US Brain Abnormalities	Mild US Brain Abnormalities
Ventriculomegaly ^1^ ≥ 15 mm	Mild ventriculomegaly ^1^ (>10 to 15 mm)
Periventricular hyperechogenicity	Intra-ventricular adhesions
Hydrocephalus ^2^	Intracranial calcifications
Microcephaly < -2DS	Subependymal cysts
Increased cisterna magna ≥ 8 mm	Choroid plexus cysts
Vermian hypoplasia	Calcifications of the lenticulostriate vessels in the basal ganglia
Porencephaly	
Lissencephaly	
Periventricular cystic lesions of the white matter	
Agenesis of the corpus callosum	

^1^ Ventriculomegaly refers to increased measurement of the lateral ventricles. ^2^ Hydrocephalus refers to tri- or quadri-ventricular dilatation in relation with microencephaly in this case.

**Table 3:** Ultrasound findings in fetal infection with CMV [25,36].

**Placental findings**	
Placentomegaly and placental calcifications	2%
**Extracerebral findings**	
Small for gestational age (SGA) or fetal growth restriction (FGR)	9%
Hyperechogenic bowel	13%
Pericardial effusion, pleural effusion, hydrops, skin edema	~1%
Ascites	4.2%
Hepatomegaly and/or splenomegaly	3.8%
Liver calcifications	1.2%
Polyhydramnios	<1%
Oligohydramnios	3.4%
**Cerebral findings**	
Microcephaly	6%
Hydrocephalus	3.6%
Ventriculomegaly	6.1%
Intracranial calcifications	6.3%
Hyperechogenic periventricular halo	3%
Subependymal cysts	1.7%
Abnormal gyration (heterotopias and polymicrogyria)	<1%

## Data Availability

No new data were created or analyzed in this study.

## Supplementary Materials

The following supporting information can be downloaded at https://www.mdpi.com/article/10.3390/jcm15103645/s1. Supplementary S1: PRISMA 2020 Checklist; Table S1: Summary of various studies utilizing US-based and MRI anomalies in fetal CMV infections [6,15,22,27,29,31,32,43,46,49,50,51,54,55,56,57,58].
