# Supplementary material for: Role of Ultrasound and Fetal MRI in the Prenatal Assessment of Congenital Cytomegalovirus Infection: A Systematic Review
Source: J Clin Med. 2026 May 9;15(10):3645. doi: 10.3390/jcm15103645 (PMC13207999; doi:10.3390/jcm15103645)
Supplement: Supplementary file 1 [file jcm-15-03645-s001.zip › jcm-4171546-supplementary-S1.pdf]

PRISMA

PRISMA 2020 Checklist

Section and Topic	Item #	Checklist item	Location where item is reported
**TITLE**			
Title	1	Role of Ultrasound and Fetal MRI in the Prenatal Assessment of Congenital Cytomegalovirus Infection: A Systematic Review.	Page 1
**ABSTRACT**			
Abstract	2	Congenital cytomegalovirus (CMV) infection is the most common congenital infection worldwide and a leading cause of neurodevelopment impairment. Prenatal imaging plays a central role in diagnosis and prognostic assessment. **Methods:** We conducted a systematic review of studies evaluating the role of ultrasound, including dedicated fetal neurosonography, and magnetic resonance imaging (MRI) in the prenatal detection and assessment of CMV infection. PubMed, Web of Science, and Scopus were searched for relevant studies, and data were synthesized focusing on detected abnormalities and the incremental diagnostic value of MRI. **Results:** Fifty-nine studies were included. Dedicated neurosonography (NSG) was the primary modality for detecting CMV-related fetal brain abnormalities, particularly ventriculomegaly and cortical malformations. Fetal MRI provided additional diagnostic information, mainly through improved visualization of the brain parenchyma, allowing better detection of white matter abnormalities, migrational disorders and posterior fossa involvement, especially when performed later in gestation. **Conclusions:** Dedicated neurosonography remains the cornerstone for the evaluation of suspected congenital fetal CMV infection. Fetal MRI represents a complementary tool that can confirm, refine or extend ultrasound findings and may improve prognostic assessment when additional or subtle brain abnormalities are suspected.	Page 1
**INTRODUCTION**			
Rationale	3	Despite the increasing use of prenatal imaging, significant variability exists in the reported diagnostic performance and prognostic value of ultrasound and fetal MRI in congenital CMV infection. Differences in imaging protocols, timing of examinations, operator expertise, and outcome assessment contribute to ongoing uncertainty in prenatal counseling. Despite the growing number of systematic reviews addressing prenatal imaging in congenital CMV infection, existing syntheses have largely focused on single imaging modalities or selected aspects of diagnostic assessment. In particular, prior reviews have typically examined either the role of ultrasonography as a screening and monitoring tool or the utility of fetal MRI in the evaluation of the central nervous system, often without direct comparison between these techniques or integration within a unified diagnostic framework. Furthermore, many of these studies have not included a systematic assessment of study quality or risk of bias. The present systematic review extends the existing body of evidence by providing a parallel evaluation of both ultrasonography and fetal MRI in the prenatal assessment of congenital CMV infection, emphasizing their complementary roles, differences in diagnostic sensitivity, and the clinical relevance of detected abnormalities. In addition, the application of a structured quality assessment enables a more critical appraisal of the available evidence and supports a more robust translation of findings into clinical practice.	Page 2/3
Objectives	4	The present systematic review extends the existing body of evidence by providing a parallel evaluation of both ultrasonography and fetal MRI in the prenatal assessment of congenital CMV infection, emphasizing their complementary roles, differences in diagnostic sensitivity, and the clinical relevance of detected abnormalities.	Page 3
**METHODS**			
Eligibility criteria	5	Predefined inclusion and exclusion criteria were established prior to the literature search. Studies were eligible for inclusion if they evaluated the role of prenatal ultrasonography, including dedicated fetal neurosonography, and/or fetal magnetic resonance imaging (MRI) in the assessment of suspected or confirmed congenital cytomegalovirus (CMV) infection. Eligible studies were required to report prenatal imaging	Page 3

Section and Topic	Item #	Checklist item	Location where item is reported
		findings in fetuses with suspected or confirmed congenital CMV infection and to provide information on cerebral and/or extracerebral abnormalities detected by ultrasound and/or MRI.<br>Studies were excluded if they focused exclusively on maternal infection, serological diagnosis, treatment, prevention, or postnatal imaging without reporting prenatal imaging findings. Review articles, editorials, conference abstracts, and case reports with insufficient imaging description were excluded. Only studies published in English between January 2003 and October 2025 were considered.	
Information sources	6	A systematic literature search was conducted using the following electronic databases: PubMed, Web of Science, and Scopus. The last search was performed on 1 October 2025.	Page 3
Search strategy	7	(("Cytomegalovirus Infections"[Mesh] OR cytomegalovirus OR CMV OR cCMV OR "congenital cytomegalovirus")<br>AND ("Ultrasonography, Prenatal"[Mesh] OR ultrasound OR ultrasonography OR neurosonography OR "prenatal ultrasound") AND ("Magnetic Resonance Imaging"[Mesh] OR MRI OR "fetal MRI" OR "magnetic resonance") AND<br>(fetus OR fetal OR foetal OR prenatal OR antenatal))	Page 4
Selection process	8	This systematic review was conducted in accordance with the Preferred Reporting Items for Systematic Reviews and Meta-Analyses (PRISMA) guideline. Study selection was performed in two stages. First, titles and abstracts were screened to exclude clearly irrelevant studies. Second, full-text articles were assessed for eligibility based on the predefined inclusion and exclusion criteria. The selection process was conducted independently, and any disagreements were resolved through discussion and consensus among the authors.	Page 4
Data collection process	9	Data extracted from the included studies comprised study characteristics (author, year, study design), population characteristics, imaging modality (prenatal ultrasound and/or fetal MRI), reported fetal brain abnormalities (e.g., ventriculomegaly, intracranial calcifications, cortical malformations, white matter abnormalities), and reported neonatal or postnatal outcomes, including sensorineural hearing loss and neurodevelopmental impairment. Given the heterogeneity of study designs, imaging protocols, and outcome measures, a qualitative synthesis of the results was performed.	Page 5
Data items	10	The primary outcomes of interest were the detection of fetal abnormalities in congenital cytomegalovirus (CMV) infection using prenatal imaging. Specifically, outcomes included the presence and type of central nervous system (CNS) abnormalities (e.g. ventriculomegaly, cortical malformations, white matter abnormalities) and extracranial abnormalities detected by ultrasound and fetal MRI. Where available, diagnostic performance measures such as sensitivity, specificity, and detection rates were extracted. All results relevant to each outcome domain reported in the included studies were collected, regardless of imaging time point or analysis method. When multiple time points or analyses were reported, the most clinically relevant prenatal assessment was prioritised.	
Study risk of bias assessment	11	The methodological quality and risk of bias of the included studies were assessed using the QUADAS-2 tool, which is specifically designed for systematic reviews of diagnostic accuracy studies. The QUADAS-2 tool evaluates four key domains: patient selection, index test, reference standard, flow and timing. The assessment was performed independently by two reviewers. Any disagreements were resolved through discussion, and if consensus could not be reached, a third reviewer was consulted. The methodological quality of the included studies substantially influences the interpretation of the findings of this review.	Page 5

PRISMA

PRISMA 2020 Checklist

Section and Topic	Item #	Checklist item	Location where item is reported
Effect measures	12	For diagnostic accuracy outcomes, effect measures included sensitivity, specificity, positive predictive value (PPV), and negative predictive value (NPV), where reported. In addition, detection rates of fetal abnormalities on ultrasound and fetal MRI were extracted and presented as proportions. Due to the heterogeneity of study designs, no summary effect measures such as risk ratios or mean differences were used.	
Synthesis methods	13a	Studies were assigned to specific syntheses based on predefined grouping criteria established in the review protocol. This included classification according to imaging modality (ultrasound, fetal MRI, or combined use) and type of reported outcomes (e.g., central nervous system abnormalities, extracranial findings). Study characteristics were tabulated and compared against these predefined categories to determine eligibility for each synthesis. Discrepancies were resolved through consensus among reviewers.	
	13b	Data from included studies were extracted as reported in the original publications. Where summary statistics were missing or inconsistently reported, no imputation or statistical conversion was performed. Instead, available data were tabulated in their original form. Due to heterogeneity in reporting formats, results were standardized descriptively to facilitate comparison across studies.	
	13c	Results were presented in structured tables summarizing study characteristics and diagnostic findings, and grouped by imaging modality. A PRISMA flow diagram illustrated study selection. No forest plots were produced due to the absence of meta-analysis.	
	13d	Due to substantial heterogeneity in study design, patient populations, imaging protocols, and outcome measures, no meta-analysis was performed. Instead, a narrative synthesis of the findings was conducted. Results were grouped according to imaging modality (ultrasound and fetal MRI) and type of detected abnormalities. This approach was chosen to allow structured comparison of diagnostic performance across heterogeneous studies that did not report directly comparable effect measures.	
	13e	Formal exploration of heterogeneity using subgroup analysis or meta-regression was not performed, as no meta-analysis was conducted. However, potential sources of heterogeneity were assessed descriptively, including differences in imaging timing, study design, and diagnostic criteria across studies.	
	13f	Sensitivity analyses were not performed due to the absence of a meta-analysis. However, the robustness of findings was considered in light of variability in study quality and methodology, as assessed using the QUADAS-2.	
Reporting bias assessment	14	No formal assessment of risk of bias due to missing results (reporting bias) was performed, as no meta-analysis was conducted. Methods such as funnel plots or statistical tests for small-study effects (e.g. Egger's test) were therefore not applicable. The potential for reporting bias across studies cannot be excluded.	
Certainty assessment	15	No formal framework, such as the GRADE approach, was used to assess the certainty of the body of evidence. Therefore, no structured assessment of confidence in the overall outcomes was performed.	
**RESULTS**			

PRISMA 2020 Checklist

Section and Topic	Item #	Checklist item	Location where item is reported
Study selection	16	PRISMA PROTOCOL<br>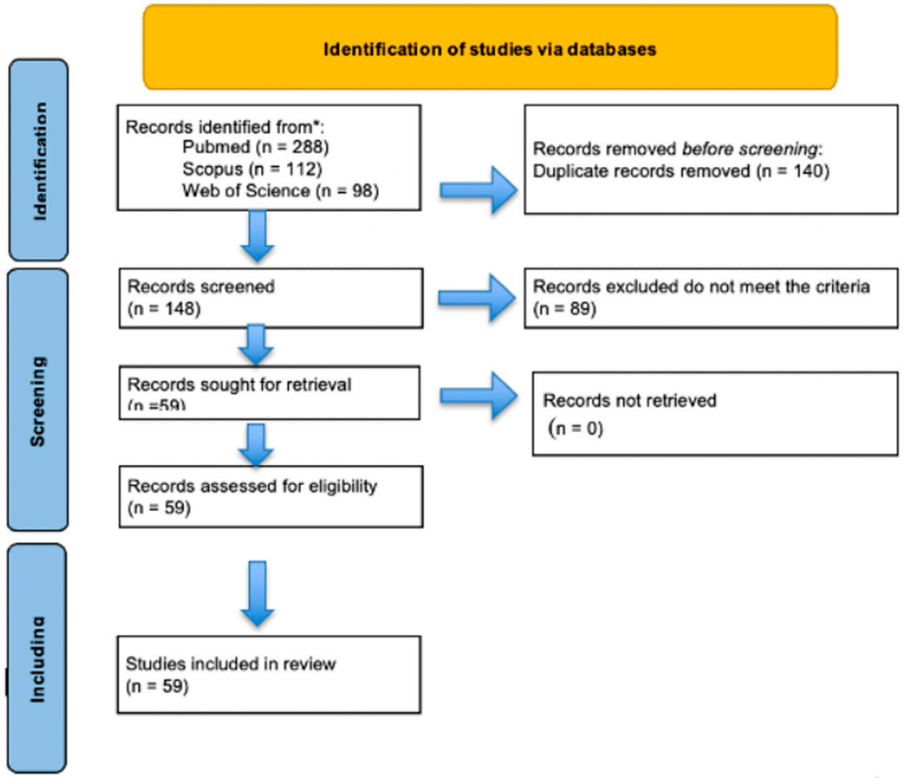<br><br>4	Page 4
Study characteristics	17	The characteristics of the included studies are presented in **SUPPLEMENTARIAL S1.**	Page 14-16
Risk of bias in studies	18	We acknowledge that a graphical summary of QUADAS-2 assessments can improve accessibility of the data. However, we have decided not to include a figure or an additional aggregated summary of the risk-of-bias assessments. Our intention was to present the results in a strictly descriptive and study-level manner, in line with the scope of this review.	-
Results of individual studies	19	Confidence intervals were not consistently reported across studies.	

Section and Topic	Item #	Checklist item	Location where item is reported
Results of syntheses	20a	Studies included in this synthesis were heterogeneous in design and methodology. According to the QUADAS-2 tool, the risk of bias was variable, with common concerns related to patient selection and study design.	
	20b	No statistical synthesis (meta-analysis) was performed due to substantial heterogeneity in study design, patient populations, imaging protocols, and outcome measures. Therefore, results are presented as a narrative synthesis.	
	20c	No formal investigation of heterogeneity (e.g., subgroup analysis or meta-regression) was performed due to the absence of a meta-analysis. However, substantial variability between studies was observed and may be explained by differences in study design, patient populations, timing of imaging, and variability in ultrasound and fetal MRI protocols.	
	20d	No sensitivity analyses were conducted, as no statistical synthesis (meta-analysis) was performed.	
Reporting biases	21	Risk of bias in individual studies was assessed using QUADAS-2. However, no formal assessment of reporting bias (arising from missing results), such as funnel plot analysis or Egger's test, was performed. Therefore, the possibility of reporting bias cannot be excluded.	
Certainty of evidence	22	Confidence in the overall body of evidence is limited by heterogeneity in imaging protocols, variability in outcome reporting, and the risk of bias identified using QUADAS-2.	
**DISCUSSION**			
Discussion	23a	Congenital cytomegalovirus infection remains a leading cause of neurodevelopmental morbidity, particularly sensorineural hearing loss, and is frequently underdiagnosed during pregnancy due to non-specific maternal presentation and limitations of routine screening strategies. Prenatal imaging plays a central role in the assessment of fetuses with suspected or confirmed CMV infection. Ultrasound represents the first-line modality, while fetal magnetic resonance imaging (MRI) provides complementary information in selected cases and may be considered as part of longitudinal follow-up in confirmed infections, particularly in specialized centers with integrated fetal MRI expertise. The prognostic value of prenatal imaging is primarily determined by the severity, extent, and progression of central nervous system abnormalities, as well as by the timing of maternal infection, rather than by isolated imaging findings. Severe and progressive prenatal imaging abnormalities are consistently associated with adverse neurodevelopmental outcomes, whereas mild or absent findings are generally associated with a more favorable prognosis, although they do not exclude the risk of delayed-onset sequelae, particularly hearing loss. Fetal MRI may offer a high negative predictive value for severe neurological impairment when no abnormalities are detected, but subtle MRI findings are associated with greater prognostic uncertainty and require cautious interpretation. Accurate prenatal counseling in congenital CMV infection should be individualized and based on an integrated, longitudinal assessment combining imaging findings, gestational timing, and virological data, with postnatal follow-up remaining essential.	Pages 19/20
	23b	Several limitations of the available evidence should be acknowledged. The reviewed studies are characterized by substantial heterogeneity in study design, imaging protocols, timing of examinations, and duration of follow-up, as well as relatively small sample sizes. In addition, long-term neurodevelopmental and auditory outcomes are not uniformly reported. These limitations highlight the need for prospective, standardized studies with comprehensive postnatal follow-up to refine risk stratification and improve evidence-based prenatal counseling.	Page 19
	23c	A limitation of this review is the inclusion of English-language publications only, which may have led to the omission of relevant studies published in other languages.	-

PRISMA 2020 Checklist

Section and Topic	Item #	Checklist item	Location where item is reported
	23d	Congenital cytomegalovirus infection remains a leading cause of neurodevelopmental morbidity, particularly sensorineural hearing loss, and is frequently underdiagnosed during pregnancy due to non-specific maternal presentation and limitations of routine screening strategies. Prenatal imaging plays a central role in the assessment of fetuses with suspected or confirmed CMV infection. Ultrasound represents the first-line modality, while fetal magnetic resonance imaging (MRI) provides complementary information in selected cases and may be considered as part of longitudinal follow-up in confirmed infections, particularly in specialized centers with integrated fetal MRI expertise. The prognostic value of prenatal imaging is primarily determined by the severity, extent, and progression of central nervous system abnormalities, as well as by the timing of maternal infection, rather than by isolated imaging findings. Severe and progressive prenatal imaging abnormalities are consistently associated with adverse neurodevelopmental outcomes, whereas mild or absent findings are generally associated with a more favorable prognosis, although they do not exclude the risk of delayed-onset sequelae, particularly hearing loss. Fetal MRI may offer a high negative predictive value for severe neurological impairment when no abnormalities are detected, but subtle MRI findings are associated with greater prognostic uncertainty and require cautious interpretation. Accurate prenatal counseling in congenital CMV infection should be individualized and based on an integrated, longitudinal assessment combining imaging findings, gestational timing, and virological data, with postnatal follow-up remaining essential.	Pages 19/20
**OTHER INFORMATION**			
Registration and protocol	24a	The protocol for this systematic review was prospectively registered in the PROSPERO database (International Prospective Register of Systematic Reviews; registration number: CRD420261339321.	Page 5
	24b	The protocol for this systematic review was prospectively registered in the PROSPERO database (International Prospective Register of Systematic Reviews; registration number: CRD420261339321 https://www.crd.york.ac.uk/PROSPERO/view/CRD420261339321	Page 5
	24c	No significant amendments were made to the protocol during the review process.	-
Support	25	No external funding was received for this study.	-
Competing interests	26	The authors declare that they have no competing interests.	-
Availability of data, code and other materials	27	The template data collection forms, data extracted from included studies, data used for all analyses, and analytic code are publicly available	-

From: Page MJ, McKenzie JE, Bossuyt PM, Boutron I, Hoffmann TC, Mulrow CD, et al. The PRISMA 2020 statement: an updated guideline for reporting systematic reviews. BMJ 2021;372:n71. doi: 10.1136/bmj.n71.
This work is licensed under CC BY 4.0. To view a copy of this license, visit https://creativecommons.org/licenses/by/4.0/
