# Supplementary material for: Role of Ultrasound and Fetal MRI in the Prenatal Assessment of Congenital Cytomegalovirus Infection: A Systematic Review
Source: J Clin Med. 2026 May 9;15(10):3645. doi: 10.3390/jcm15103645 (PMC13207999; doi:10.3390/jcm15103645)
Supplement: Supplementary file 1 [file jcm-15-03645-s001.zip › jcm-4171546-Table S1.pdf]

Table S1. Summary of various studies utilizing US-based and MRI anomalies in fetal CMV infections.

Author and Year of Publication	Study Design	Study Population (n)	Gestational Age at Imaging	Imaging Modality Used	Main Prenatal Imaging Findings	Outcome Assessed
Lipitz S et al. [29] (2010)	Prospective cohort study	38	1T, 2T, 3T	US + MRI	Echogenic bowel, periventricular cysts in caudate nucleus.	Prenatal findings, postnatal outcome, neurodevelopmental follow-up
Malinger G et al. [54] (2003)	Retrospective cohort study	8	2T, 3T	US + MRI	Periventricular echogenicity, ventriculomegaly, intraparenchymal foci, abnormal patterns of gyre and sulci, anomalies of corpus callosum, abnormalities of cerebral and cisternal magna, and vasculopathy of striatal artery	Prenatal findings, postnatal outcome, neurodevelopmental follow-up
O'Sullivan C et al. [55] (2017)	Case report	1	2T	US	Echogenic bowel, bilateral ventriculomegaly, periventricular hyperechogenicity	Prenatal findings
Minsart AF et al. [31] (2020)	Retrospective cohort study	84	N/A	US + MRI	Extracerebral anomalies, changes in neurological tissues	Prenatal findings, postnatal outcome, neurodevelopmental follow-up
Simonazzi G et al. [56] (2010)	Prospective cohort study	218	2T	US + neurosonography	Periventricular echogenic halo with well-defined borders, white matter lesions	Prenatal findings
Birnbaum R et al. [46] (2017)	Retrospective cohort study	81	1T, 2T, 3T	Neurosonography + MRI	Ventriculomegaly, periventricular or porencephalic cysts, periventricular increased echogenicity, occipital horn	Prenatal findings, postnatal outcome, neurodevelop

					cavitation, brain atrophy/destruction, cerebellar and callosal abnormalities	mental follow-up
Birnbaum R et al. [15] (2021)	Retrospective cohort study	22	1T, 2T, 3T	Neurosonography + MRI	Focal changes in germinal matrix (GM), lenticulostriate vasculopathy (LSV)	Prenatal findings, postnatal outcome, neurodevelopmental follow-up
Buca D et al. [27] (2021)	Systematic review	2603	1T, 2T, 3T	US + neurosonography + MRI	Intra-CNS and extra-CNS anomalies	Prenatal findings, postnatal outcome, neurodevelopmental follow-up
Krajden Haratz K et al. [32] (2025)	Retrospective cohort study	72	1T, 2T, 3T	US + neurosonography + MRI	Increased periventricular echogenicity, intraparenchymal calcifications, callosal abnormalities	Prenatal findings, postnatal outcome, neurodevelopmental follow-up
Hawkins-Villarreal A et al. [57] (2023)	Retrospective cohort study	35	2T, 3T	Neurosonography + MRI	Halo sign (presence of homogeneous periventricular echogenicity observed in all three fetal brain orthogonal planes), corpus callosum agenesis/dysgenesis, cerebellar and/or vermian hypoplasia	Prenatal findings, postmortem histopathology
Di Mascio D et al. [43] (2023)	Retrospective cohort study	95	2T, 3T	Neurosonography + MRI	Malformations of cortical development, destructive encephalopathy, intracranial calcifications in the germinal matrix, complex CNS anomalies in 30.0%	Prenatal findings
Sadan OR et al. [6] (2025)	Retrospective cohort study	44	3T	MRI + MRS	Infected fetuses with gross MRI findings exhibited significantly reduced tNAA/tCr	Prenatal findings

					ratios compared to the other groups	
Aertsen M et al. [22] (2022)	Retrospective cohort study	46	3T	Neurosonography + MRI	Temporal cysts, abnormal gyration, periventricular calcifications, lenticulostriate vasculopathy	Prenatal findings
Di Mascio D et al. [58] (2021)	Retrospective cohort study	187	2T, 3T	Neurosonography + MRI	US: isolated ventriculomegaly, MRI: malformations of cortical development and midline anomalies	Prenatal findings
D'Amico A et al. [49] (2021)	Systematic review	12971	1T, 2T, 3T	US	Echogenic bowels	Prenatal findings, postnatal outcome
Guerra B et al. [50] (2008)	Retrospective cohort study	650	2T, 3T	US	Choroid plexus cysts, mild unilateral pyelectasis, hyperechogenic bowel, ventriculomegaly and enlargement of cisterna magna	Prenatal findings, postnatal outcome, neurodevelopmental follow-up
Imafuku H et al. [51] (2020)	Prospective cohort study	4380	N/A	US	Ventriculomegaly, intracranial calcification, hyperechogenic bowel, microcephaly, hepatosplenomegaly and ascites.	Prenatal findings, postnatal outcome
